# Baths and Bathing

**Published:** 1880-09

**Authors:** 


### BATHS AND BATHING.

A cold bath is in water under seventy degrees Fahrenheit, a warm one, is over ninety. A warm bath in pure water debilitates, in salt water, strengthens.

